# A pan-cancer analysis reveals the genetic alterations and immunotherapy of Piezo2 in human cancer

**DOI:** 10.3389/fgene.2022.918977

**Published:** 2022-08-04

**Authors:** Xin Liu, Yangpu Jia, Zhihui Wang, Zhaoxiong Zhang, Weihua Fu

**Affiliations:** ^1^ Department of General Surgery, Tianjin Medical University General Hospital, Tianjin, China; ^2^ Department of Gastrointestinal Surgery, Wuhan Central Hospital, Tongji Medical College, Huazhong University of Science and Technology, Wuhan, China

**Keywords:** Piezo2, pan-cancer, prognostic, genetic alterations, tumor microenvironment

## Abstract

**Background:** Piezo2 is a transmembrane-spanning ion channel protein implicated in multiple physiological processes, including cell proliferation and angiogenesis in many cell types. However, Piezo2 was recognized as representing a double-edged sword in terms of tumor growth. The prognostic and immunotherapeutic roles of Piezo2 in pan-cancer have not been reported.

**Methods:** In this study, several databases available including the UCSC Xena database, HPA, TIDE, GSEA, and cBioportal were used to investigate the expression, alterations, associations with immune indicators, and prognostic roles of Piezo2 across pan-cancer. R software and Perl scripts were used to process the raw data acquired from the UCSC Xena database.

**Results:** Based on processed data, our results suggested that Piezo2 expression levels were tissue-dependent in different tumor tissues. Meanwhile, the survival analysis reflected that patients suffering from KIRC, LUAD, and USC with high Piezo2 expression had good OS, while those suffering from KIRP and SARC with high Piezo2 expression had poor OS. In addition, our results showed that Piezo2 expression was associated with the infiltration of CD4^+^ T memory cells, mast cells, and dendritic cells. These results suggested that Piezo2 may involve tumor progression by influencing immune infiltration or regulating immune cell function. Further analysis indicated that Piezo2 could influence TME by regulating T-cell dysfunction. We also found that gene mutation was the most common genetic alteration of Piezo2. The GSEA analysis revealed that Piezo2 was associated with calcium ion transport, the activation of the immune response, antigen processing and presentation pathways.

**Conclusion:** Our study showed the expression and prognostic features of Piezo2 and highlighted its associations with genetic alterations and immune signatures in pan-cancer. Moreover, we provided several novel insights for further research on the therapeutic potential of Piezo2.

## Introduction

As a common and frequently-occurring disease at present, cancer seriously threatens human health, reduces the quality of life, and brings a heavy economic burden to patients (Sung et al., 2021). With in-depth studies of cancer, we know that cancer initiation and progression are accompanied by multiple accumulations of genetic alterations, epigenetic alterations (Dawson and Kouzarides, 2012), cell metabolism (Justus et al., 2015), and cancer microenvironment changes (Wang et al., 2018). A growing body of evidence demonstrated that genetic alterations could drive genome instability and mutation, a hallmark of cancer. Hence, it is necessary to figure out the associations between genetic alterations and genome variation.

The tumor microenvironment (TME) mainly consists of tumor cells and various tissue components, including cancer-associated fibroblasts (CAF), microvessels, immune cells, cytokines, and chemokines (Wu and Dai, 2017)et al. Among them, immune cells, an essential component of TME, are recruited to the ecological niche in response to signals released by tumor cells. The anti-tumor effector functions of immune cells in TME were hampered or reversed into pro-tumor effector functions (Whiteside, 2008). *Cancer* cells implemented proliferation, migration, and invasion by escaping from immune surveillance. Thus, current cancer therapy is mainly focused on maintaining immune homeostasis and restoring immune surveillance by reactivating the capacity of immune cells (Galluzzi et al., 2020). Recently, emerging therapeutic targets and potential pathologic mechanisms have gradually been revealed. Many drugs targeting immune checkpoints, including PD-1, CTLA-4, TIM3, etc., have been approved for clinical application (Rotte, 2019). A study also has shown that angiopoietin-2 in melanoma was a potential predictive and prognostic biomarker for immune checkpoint therapy (Wu et al., 2017). Unfortunately, immune checkpoint inhibitors were effective in improving patient survival rates with multiple cancers, but they might bring adverse events to patients. And many patients still respond poorly to these therapies currently available. It is still essential to find novel targets for immune therapy and overcoming immune therapy resistance.

Piezo2, a member of the piezo family, is a cation-selective mechanical channel and mediates cation influx in which calcium ions (Ca^2+^) predominate (Coste et al., 2010). Piezo1 and Piezo2 played significant roles in regulating diverse physiological processes including sensing tactile sensations (Wang et al., 2019), regulating blood pressure (Zeng et al., 2018), prompting vascular development (Yang et al., 2016), and conducting mechanical force (Coste et al., 2010) et al. In addition to these, increasing shreds of evidence demonstrated that piezo protein could influence cancer development, progression, and invasion. During the past years, Piezo1 has been widely studied in cancers. Some studies have confirmed that Piezo1 could promote malignant biological behavior involving viability, migration, and metastasis of colon cancer cells (Sun et al., 2020; Jiang et al., 2021). Moreover, Piezo1 has been identified as a cancer-promoting factor in a variety of cancers, including hepatocellular carcinoma (Liu et al., 2021), gastric cancer (Wang et al., 2021), oral squamous cell carcinoma (Hasegawa et al., 2021), and breast cancer (Luo et al., 2022). However, the functional role of Piezo2 has not received much attention in cancers (Yang et al., 2016). Suggested that Piezo2 expression was elevated in glioma tumor cells, and Piezo2 downregulation inhibited the proliferative capacity of tumor cells and reduced tumor angiogenesis. As well, it has also been revealed that Piezo2 was associated with the progression of laryngeal squamous cell carcinoma and was a promising biomarker used in diagnosis and prognosis (Cheng et al., 2017). In contrast, a study has shown that Piezo2 expression levels were downregulated in non-small cell lung cancer (NSCLC) compared with normal tissues. Meanwhile, high Piezo2 expression was associated with better overall survival (OS) for patients (Huang et al., 2019). Likewise, a previous study reported that decreased expression of Piezo2 predicted poor prognosis of patients with breast cancer (Lou et al., 2019). Thus, Piezo2 might exert cancer-promoting or cancer‐suppressing effects in various cancers. It is, therefore, essential to explore the specific roles of Piezo2 in pan-cancer.

In the present study, we performed a comprehensive analysis to investigate the expression, genetic alteration, associations with immune indicators, and prognostic roles of Piezo2 across 33 cancers using the UCSC Xena, HPA, TIDE, GSEA, and cBioportal databases. Our study may further broaden the cancer landscape of Piezo2 and provide novel insights into cancer therapy.

## Materials and methods

### Data collection and processing

**FIGURE 1:**
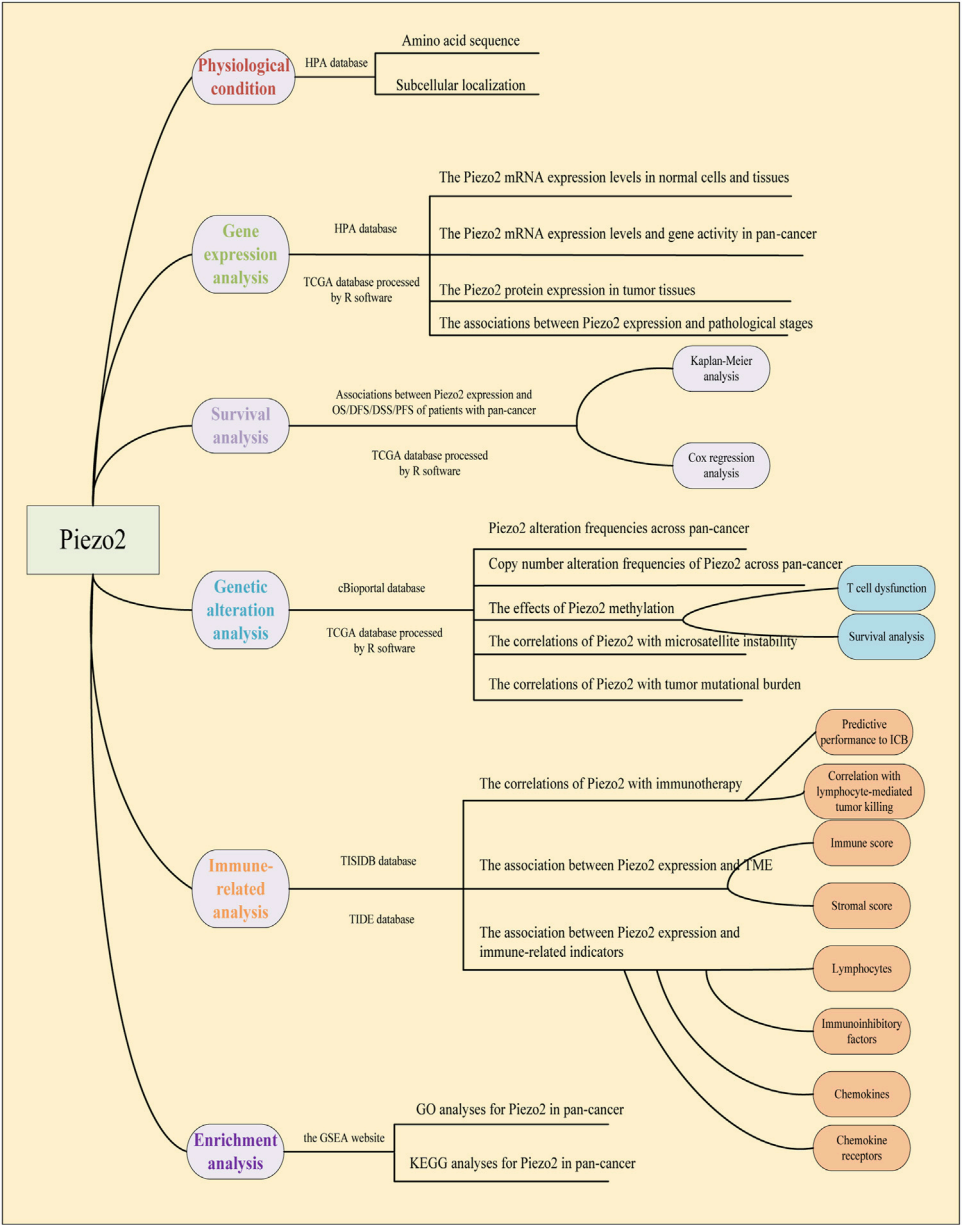
Workflow of pan-cancer analysis of Piezo2.

The gene expression data, clinical phenotype data, and mutation data of 33 cancers were downloaded from the UCSC Xena database (https://xena.ucsc.edu/). The database contained transcriptomic data of tumor samples and normal samples. The raw data were processed for ID conversion and other format conversion steps by R software (https://www.R-project.org) and Strawberry Perl. RNAseq data in FPKM format was converted into TPM format and log2 conversion was performed. The workflow of our study was shown in Figure 1.

### The human protein atlas database

HPA (http://www.proteinatlas.org) is an open-access resource used for analyzing protein expression in tissues, single-cell types, immune cells, cell lines, and other subcellular levels. The protein subcellular localization, expression levels in cell lines, and expression levels in tissues of Piezo2 were obtained from the HPA database. The subcellular localization of Piezo2 was analyzed in the Hela cell line, Hep G2 cell line, and U-2 OS cell line. The relationship between the expression of Piezo2, endoplasmic reticulum (ER), microtubules, and nucleus was explored in the HPA database.

### Relationships with pathological staging and prognostic value of Piezo2 in pan-cancer

Piezo2 mRNA expression differences and gene activity differences in 33 cancers were analyzed using “lima” and “GSVA” packages and visualized by “ggpubr” packages in R software. The gene activity scores of Piezo2 were measured by ssGSEA. The Kaplan-Meier method and univariate Cox regression analysis were used for survival analyses including OS, disease-free survival (DFS), disease-specific survival (DSS), and progression-free survival (PFS) across various cancers. Data analysis and graphical output were performed by “survival”, “survminer”, and “forestplot” packages.

To comprehensively evaluate the relationship between Piezo2 and clinical staging, we used the TCGA database and made a pathological stage plot of different cancer types. And the relationships between Piezo2 and T stage, N stage, and M stage were further analyzed separately. The data was processed and visualized by the “ggpubr” package in R software.

### Analysis of the correlations between Piezo2 expression and the immune microenvironment in pan-cancer

The immune scores and stromal scores of samples were obtained using the ESTIMATE algorithm (Yoshihara et al., 2013) in R software. Calculation of immune scores and stromal scores were based on the different expression levels of specific genes in different immune and stromal cells. The correlation analysis between Piezo2 expression and immune/stromal scores was performed by “ggplot2”, “ggpubr”, and “ggExtra” packages. The relationships between Piezo2 expression and immune-related indicators including lymphocytes, immunoinhibitory, chemokines, and chemokine receptors were analyzed using the TISIDB database (http://cis.hku.hk/TISIDB/index.php).

### Gene ontology and kyoto encyclopedia of genes and genomes

The GO and KEGG gene annotation files were downloaded from the GSEA website (http://www.gsea-msigdb.org/). According to the expression levels of Piezo2, the samples were divided into two groups with high and low expression and then matched with GO and KEGG annotation files using “limma”, “clusterProfiler”, “enrichplot”, and “ggplot2” packages in R software. The top 5 pathways and functions were displayed.

### Analysis of epigenetic alterations

The data of tumor mutational burden (TMB) and microsatellite instability (MSI) in pan-cancer were downloaded as mentioned above and then integrated by Strawberry Perl. The correlations between Piezo2 expression and TMB/MSI were analyzed using the Spearman method, then visualized by the “fmsb” package in R software. The Piezo2 copy number alterations and genetic alterations in different cancers were analyzed by the cBioportal database (http://www.cbioportal.org/).

### The tumor immune dysfunction and exclusion database

Tumor Immune Dysfunction and Exclusion (TIDE) database (Fu et al., 2020), which contained 33,197 samples and 189 cancer studies, was a predictor of immune checkpoint blockade therapy (http://tide.dfci.harvard.edu/). The Piezo2 predictive performance was calculated in TIDE compared with some published predictive markers in immune checkpoint blockade (ICB) cohorts. The associations between Piezo2 expression with ICB response outcomes and T cell dysfunction levels were evaluated in diverse cohorts. And then, the roles of Piezo2 methylation in T cell dysfunction and its risk scores were explored in various cancers.

### Statistical analyses

All statistical analyses were performed using R (4.1.0) software and the abovementioned R packages. The statistical analyses were analyzed by Student’s t-test or ANOVA when appropriate. The Cox regression model and Kaplan-Meier method were used to performing survival analysis. *p* < 0.05 was considered statistically significant if not particularly specified.

## Results

### Piezo2 localization, composition, and expression of in normal, neoplastic cell lines and tissues

**FIGURE 2:**
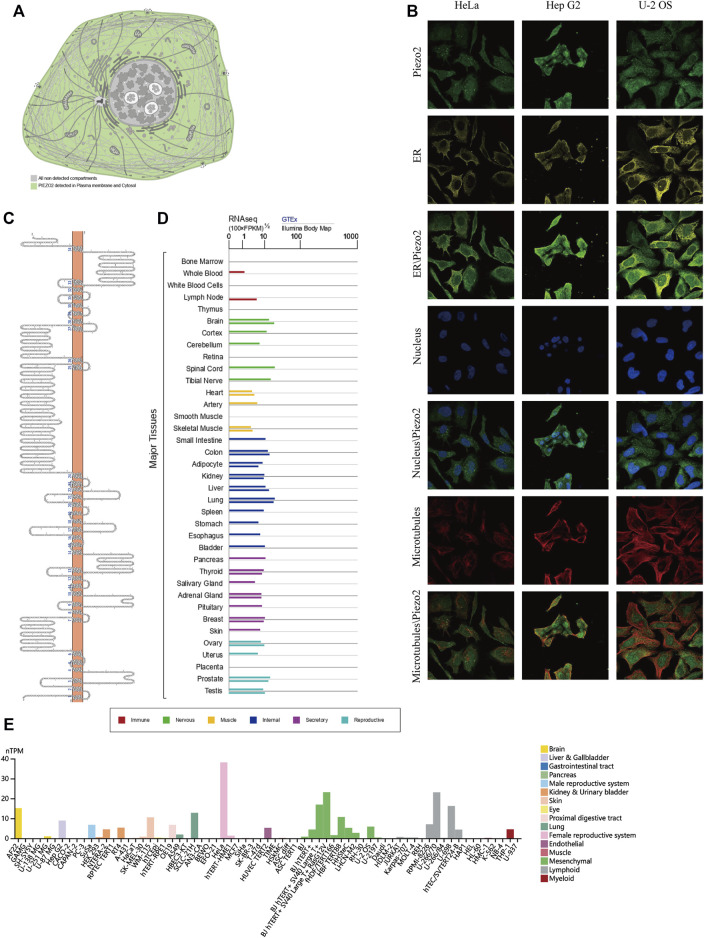
The localization, composition, and expression levels of Piezo2 in normal cells and tissues. **(A)** The expression of Piezo2 was mainly located at the plasma membrane and cytoplasm. **(B)** The expression and localization analyses of Piezo2, endoplasmic reticulum (ER), microtubules, and nucleus in Hela, Hep G2, and U-2 OS by immunofluorescence as adopted from the HPA database. **(C)** The protein topology structure of Piezo2. The structure showed the association between the protein structure of Piezo2 and the cell membrane. **(D)** Piezo2 mRNA expression levels in various normal tissues. The mRNA of Piezo2 was expressed in similar levels in most tissues. **(E)** Piezo2 mRNA expression levels were different in various tumor cell lines.

The essential functions of protein were always associated with its localization, structure, and sequence (Whisstock and Lesk, 2003). Most methods used in predicting protein function always rely on contrasting amino acid sequences and spatial structures with known proteins. To explore the intracellular protein localization of Piezo2, we retrieved its protein expression and distribution in the HPA database. Piezo2 was mainly detected in plasma membrane and cytoplasm, as shown in the simulation diagram (Figure 2A). As shown in Figure 2B, the distribution of Piezo2 was co-localized with the ER, microtubules, and nucleus in Hela, Hep G2, and U-2 OS cells. The protein topology structure of Piezo2 suggested that Piezo2 had 34 transmembrane domains which were the same as described in the literature (Figure 2C). Furthermore, we assessed the mRNA expression levels of Piezo2 in various normal tissues, including the digestive system, nervous system, cardiovascular system, etc., and multiple human tumor cell lines. We found that Piezo2 was widely expressed in a wide variety of normal human tissues and tumor cell lines (Figures 2D,E).

### The mRNA and protein expression of Piezo2 in pan-cancer

**FIGURE 3:**
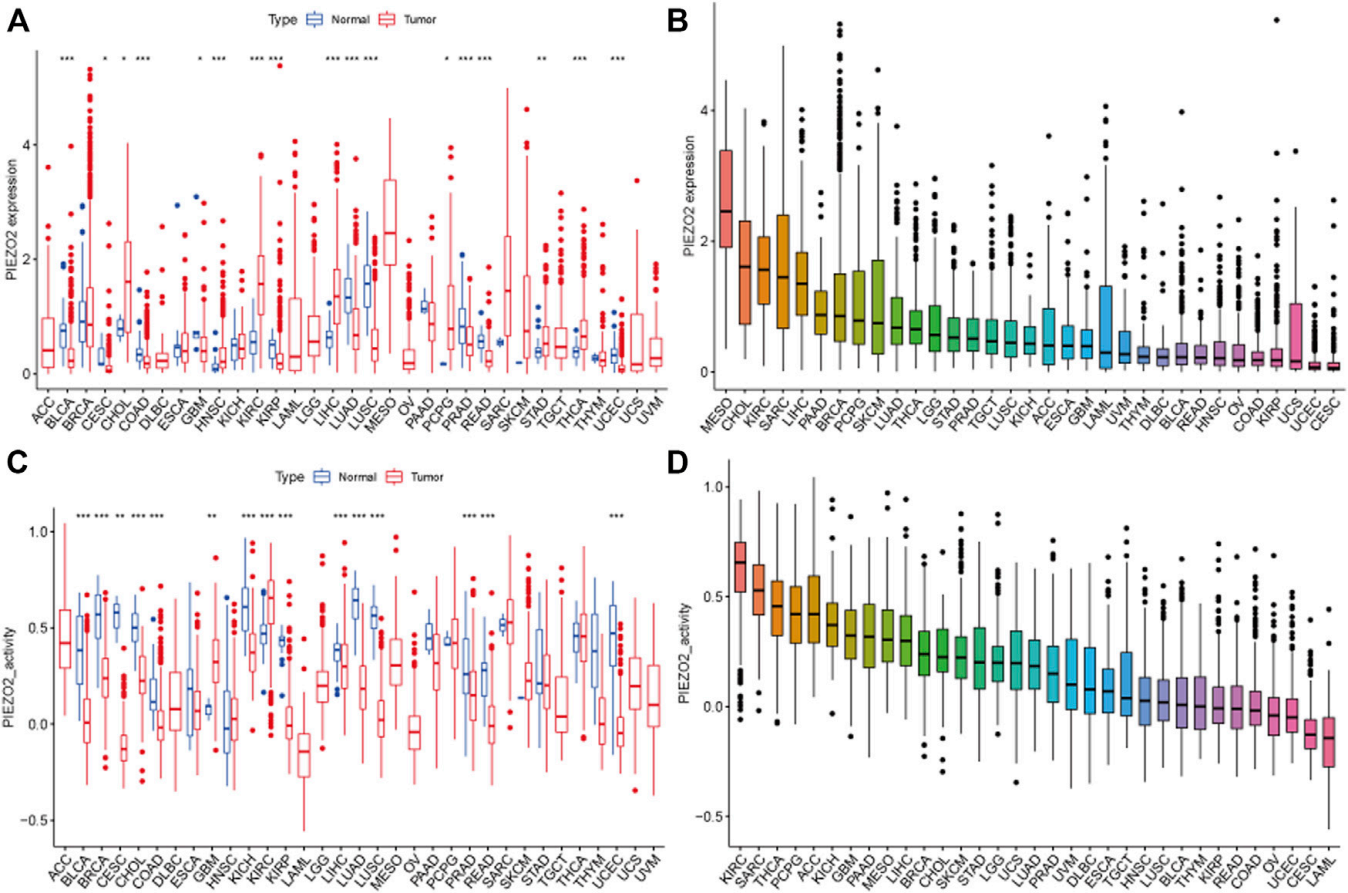
Piezo2 expression levels and gene activity in 33 cancers. **(A)** Expression differences of Piezo2 in diverse cancers compared with normal tissues. Among them, Piezo2 was upregulated in CHOL, HNSC, KIRC, LIHC, PCPG, STAD, and THCA compared with normal tissues. In BLCA, COAD, GBM, KIRP, LUAD, LUSC, PRAD, and READ, Piezo2 was down-regulated in tumor tissues. **(B)** Relative expression differences of Piezo2 in diverse cancers. **(C)** Gene activity of Piezo2 in diverse cancers compared with normal tissues. Piezo2 had higher activity in tumor tissues of GBM, and KIRC. In BLCA, BRCA, CESC, CHOL, COAD, KICH, KIRP, LIHC, LUAD, LUSC, PRAD, READ, and UCEC, the gene activity of Piezo2 was higher in normal tissues. **(D)** Gene activity differences of Piezo2 in diverse cancers. *p < 0.05, **p < 0.01, ***p < 0.001.

To identify the associations between Piezo2 and cancers, we explored the expression differences of Piezo2 in tumor tissues. *Cancer* type abbreviations were shown in Supplementary Table S1. The mRNA expression levels of Piezo2 in tumor tissues were higher than in normal tissues in CHOL, HNSC, KIRC, LIHC, PCPG, STAD, and THCA (*p* < 0.05). On the contrary, Piezo2 expression levels in tumor tissues were lower than in normal tissues in BLCA, CESC, COAD, LUAD, etc. (Figure 3A). We sorted the expression levels of Piezo2 in tumor tissues and found that Piezo2 expression levels were the highest in MESO and lowest in CESC (Figure 3B). Furthermore, we evaluated the activity of Piezo2, which suggested that the gene activity of Piezo2 was grossly consistent with the trend of expression level (Figure 3C). Interestingly, the analysis results of Piezo2 gene activity were not the same as gene expression levels in different tumors (Figure 3D). Piezo2 gene activity was highest in KIRC and lowest in LAML. These results suggested that Piezo2 may have different co-expressed genes in different cancers. Further analysis of protein expression of Piezo2 in tumors showed that Piezo2 protein was also expressed in various tumor tissues, including the cerebral cortex, kidney, stomach, etc (Supplementary Figure S1).

### Relationship between Piezo2 and clinical staging in pan-cancer

**FIGURE 4:**
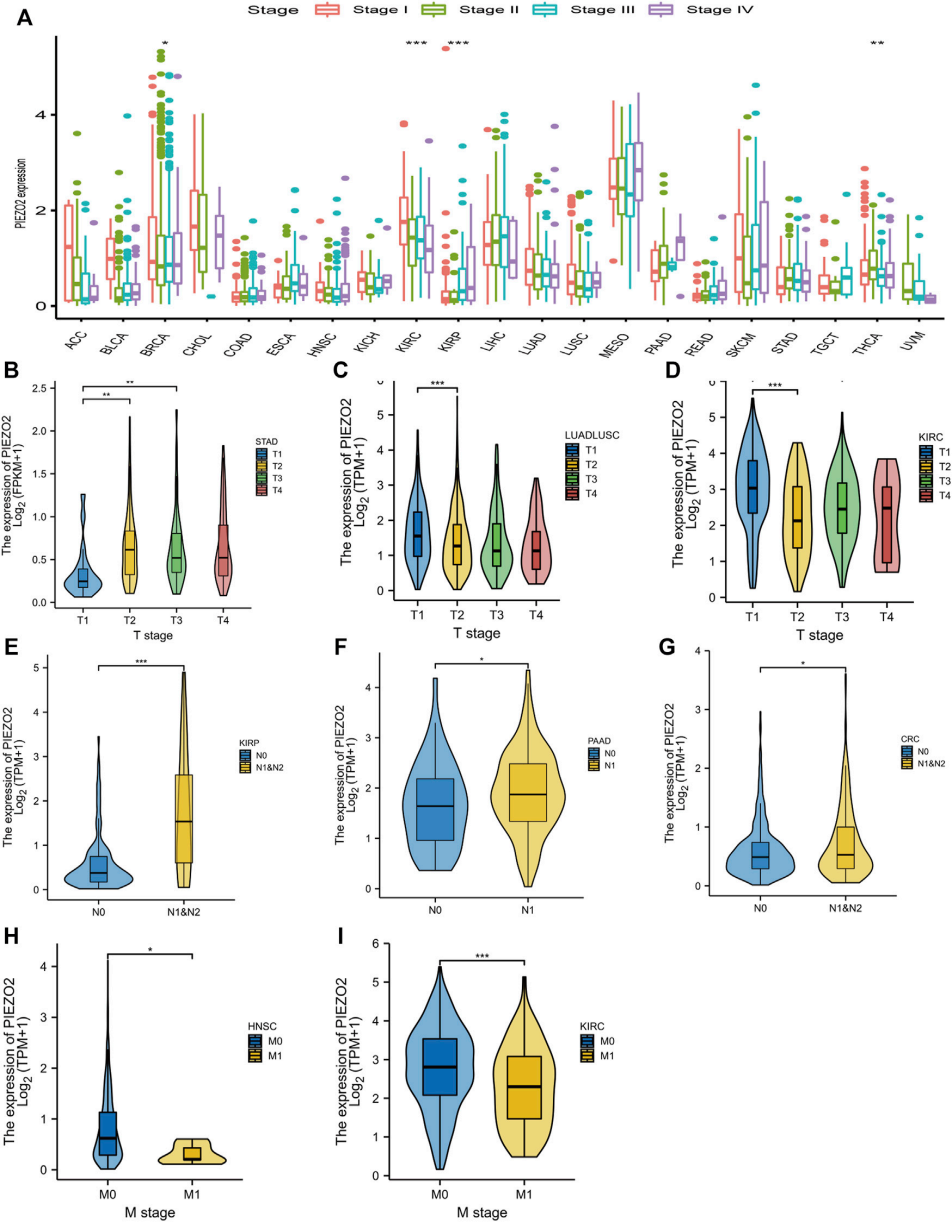
The relationship between Piezo2 expression levels and tumor stage. **(A)** The relationship between Piezo2 mRNA expression levels and tumor stage in pan-cancer. **(B–D)** The relationship between Piezo2 mRNA expression levels and tumor size in STAD, LUADLUSC, and KIRC. **(E–G)** The relationship between Piezo2 mRNA expression levels and lymph node metastasis in KIRP, PAAD, and CRC. **(H,I)** The relationship between Piezo2 mRNA expression levels and distant metastasis in HNSC and KIRC. *p < 0.05, **p < 0.01, ***p < 0.001.

Owing to the abnormal expression of Piezo2 in tumor tissues, we accessed the relationship between Piezo2 expression and the clinical staging of tumors (Figure 4). As shown in Figure 4A, Piezo2 expression levels were associated with different clinical staging in BRCA, KIRC, KIRP, and THCA. We also analyzed the associations between Piezo2 expression and T, N, and M stages in depth. There was no apparent association between Piezo2 expression and tumor size in different tumors (Figures 4B–D). Meanwhile, we noticed that Piezo2 was upregulated in tumors with lymph node metastasis (Figures 4E–G). However, Piezo2 was down-regulated in tumors that occurred with distant metastases (Figures 4H,I).

### Correlations between Piezo2 and prognosis in pan-cancer

**FIGURE 5:**
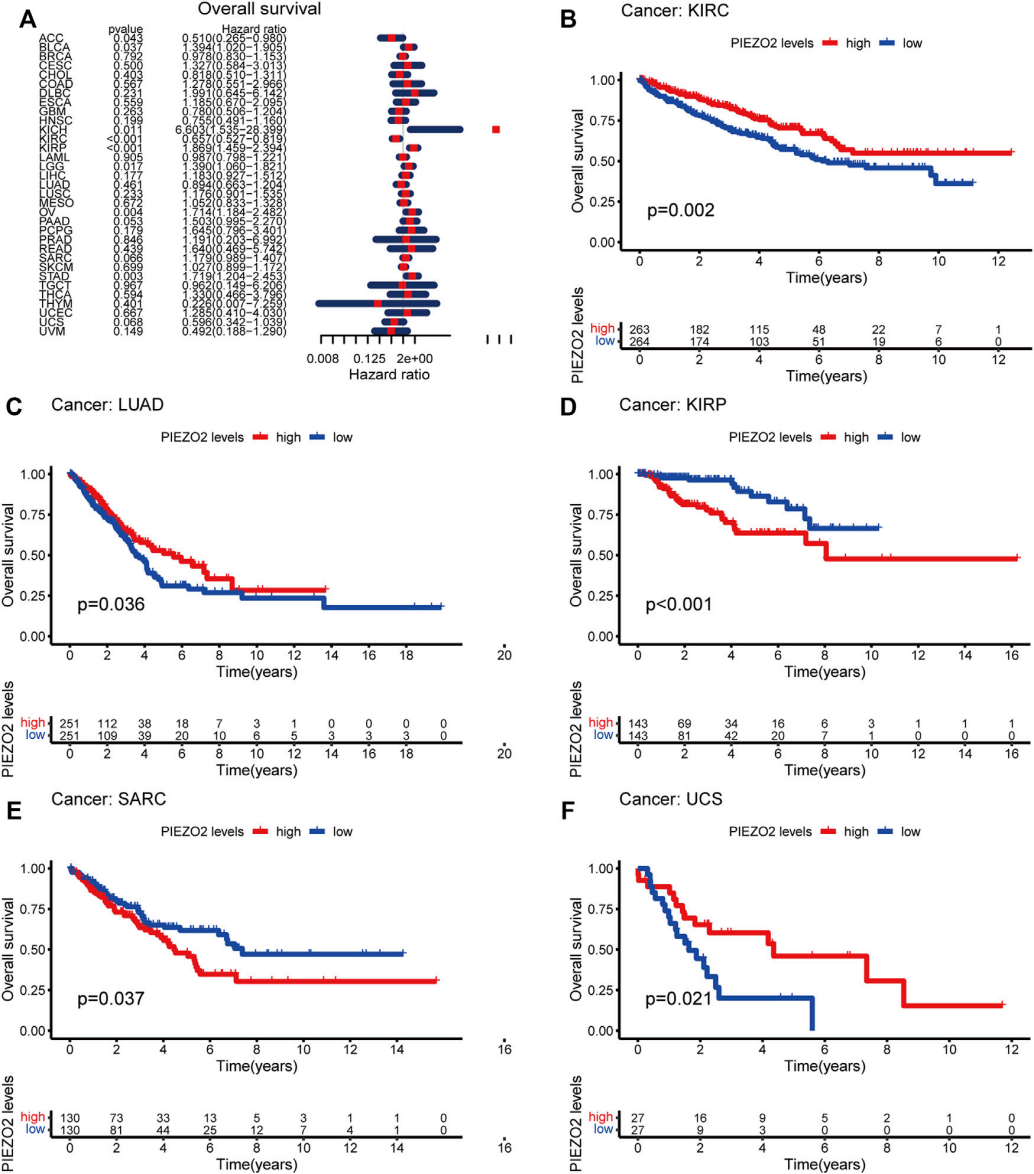
Piezo2 expression levels were associated with OS in multiple cancers. **(A)** Cox regression of Piezo2 and OS in pan-cancer. **(B–F)** Kaplan-Meier analysis of Piezo2 expression and OS in KIRC, LUAD, KIRP, SARC, and UCS.

**FIGURE 6:**
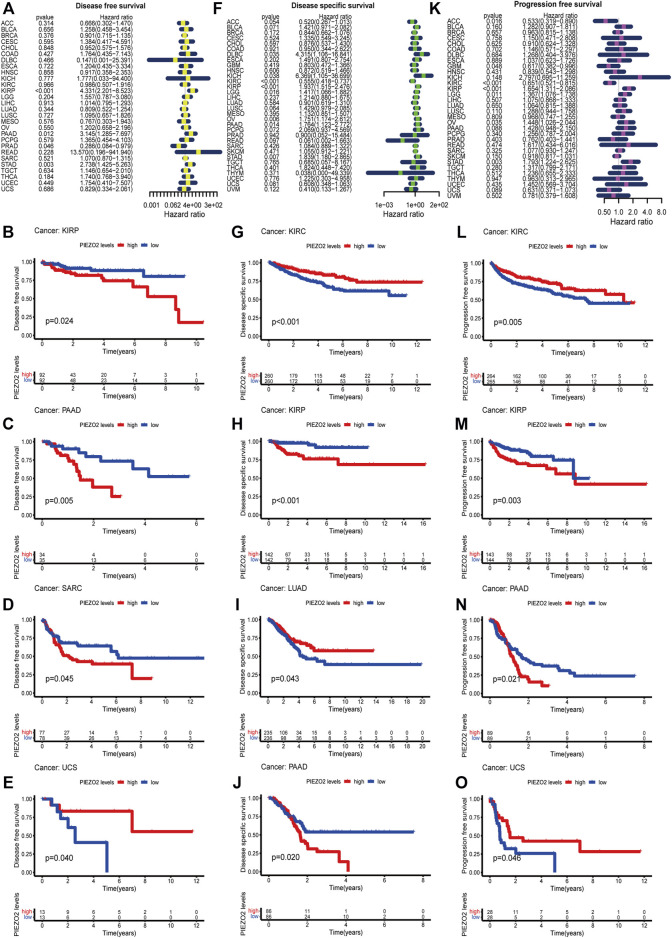
Piezo2 expression levels were associated with DFS, DSS, and PFS. Cox regression and Kaplan-Meier analysis of Piezo2 with DFS **(A–E)**, DSS **(F–J)**, and PFS **(K–O)**.

We further accessed the impact of Piezo2 expression on the prognosis of patients with multiple cancers. The survival analysis was verified by Cox regression and Kaplan-Meier analysis, respectively. The two methods were combined to explore the impact of Piezo2 expression on patient prognosis. As shown in Figures 5, 6, the relationship between the expression levels of Piezo2 and prognosis was not consistent in different cancers. The results of OS showed that Piezo2 was a protective factor in KIRC, LUAD, and UCS and an indicator of poor prognosis in KIRP and SARC (Figures 5A–F). The analyses of DFS revealed that Piezo2 acted as an unfavorable factor in KIRP, PAAD, and SARC (Figures 6A–E). Furthermore, the DSS analyses revealed that Piezo2 acted as a protective factor in KIRC, and LUAD and a risk factor in KIRP and PAAD (Figures 6F–J). We also explored whether Piezo2 expression affected the PFS of cancer patients. The results showed that increased Piezo2 expression was associated with a good prognosis for patients with KIRC and UCS (Figures 6K–O). While the PFS of patients from KIRP and PAAD with higher Piezo2 expression was shorter than those with lower expression. Thus, Piezo2 may play both cancer-promoting or cancer-suppressing functions in different tumor types.

### Associations between Piezo2 and immune-related indicators

**FIGURE 7:**
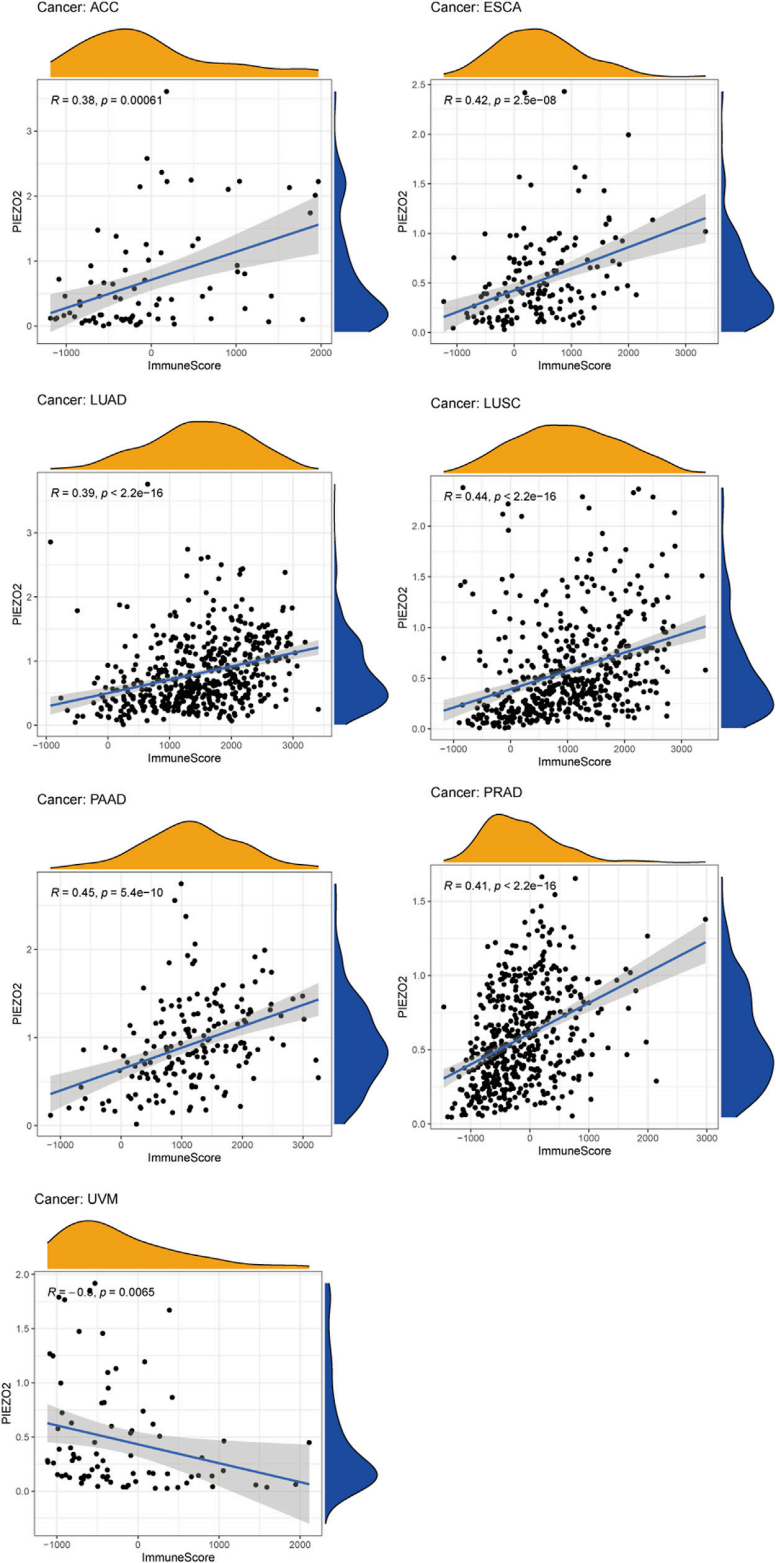
The associations between Piezo2 expression and immune scores in multiple cancers. Only results with *p* < 0.05 and |R| > 0.3 were considered for analysis.

**FIGURE 8:**
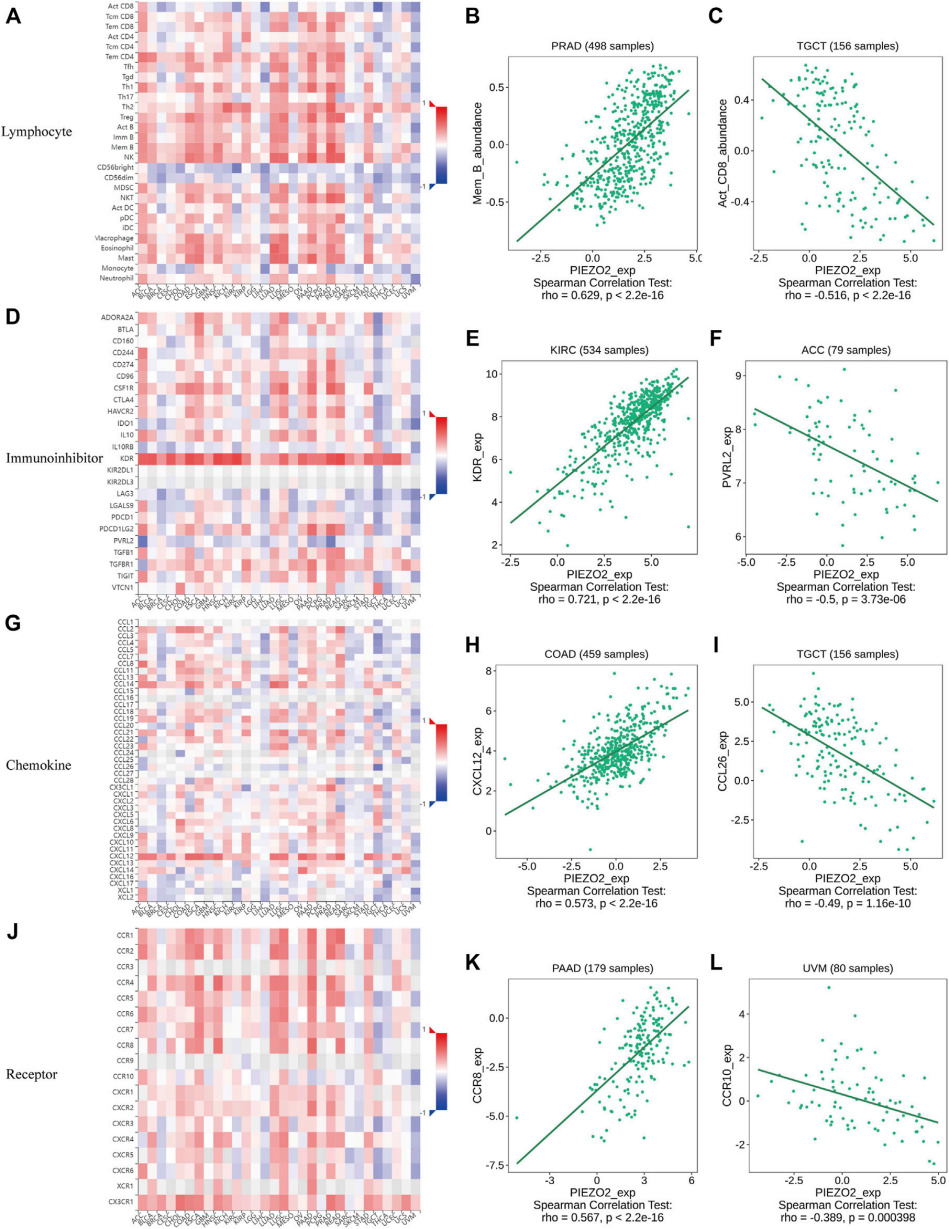
Heatmap and scatter plot showed the correlations of Piezo2 expression with immune-related indicators, including lymphocytes **(A–C)**, immunoinhibitory factors **(D–F)**, chemokines **(G–I)**, and chemokine receptors **(J–L)**. The strongest representative of the positive and negative correlations of the indicators was shown in scatter plots.

Growing evidence indicated that the tumor immune microenvironment may play an important role in the maintenance and progression of tumors. Hence, in the current study, we explored the associations between Piezo2 expression and immune signatures by scoring immune-related genes and stromal-related genes (Figure 7, Supplementary Figure S2). Overall, the associations between Piezo2 expression and immune/stromal scores showed a positive correlation. Among them, the strongest observed correlation between immune scores and Piezo2 was in PAAD (*r* = 0.45, *p* = 5.4e-10). And the strongest observed correlation between stromal scores and Piezo2 was in PRAD (*r* = 0.69, *p* < 2.2e-16). To further analyze whether Piezo2 can affect the immune environment, we explored the associations with other immune indicators, including lymphocytes, immunoinhibitory factors, chemokines, and chemokine receptors in TIBSD (Figure 8). The strongest indicators of positive and negative correlations were exhibited. Surprisingly, Piezo2 showed a positive correlation with memory B cells in PRAD (*r* = 0.629) and a negative correlation with activated CD8^+^ cytotoxic cells in TGCT (*r* = −0.516) (Figures 8A–C). In an analysis of immunoinhibitory factors, Piezo2 showed a strong positive correlation with KDR in numerous immunosuppressive factors (Figures 8D–F). And in the correlation analysis between Piezo2 expression and chemokine, Piezo2 showed the positively strongest correlation with CXCL12 (*r* = 0.573) in COAD and the negatively strongest correlation with CCL26 (*r* = −0.49) in TGCG (Figures 8G–I). In the observation of chemokine receptors, Piezo2 had the strongest positive correlation with CCR8 (*r* = 0.567) in PAAD and the strongest positive correlation with CCR10 (*r* = −0.389) in UVM (Figures 10J–L).

### Epigenetic alterations of Piezo2 and its associations with immunotherapy of cancers

**FIGURE 9:**
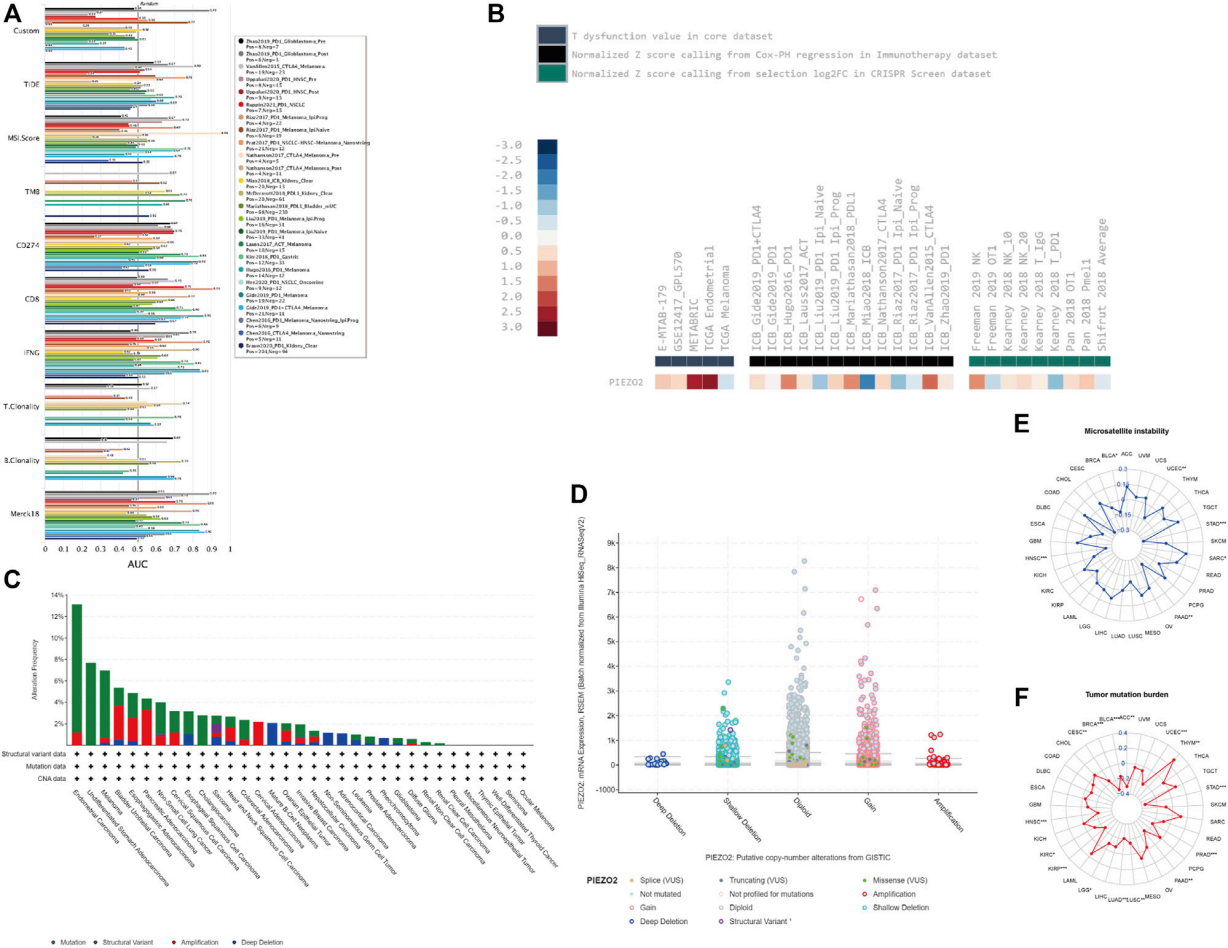
Piezo2 genetic alterations and associations with tumor immunity responses. **(A)** The bar chart of the receiver operating characteristic curve (AUC) showed the relevance of Piezo2 compared with published tumor immune evasion biomarkers. The size of the AUC value reflected predictive performances of the garget markers on the immune checkpoint blockade. “Custom” represented our target gene “Piezo2”. **(B)** The analyses of Piezo2 associations with T-cell dysfunction, outcomes using ICB treatment, and lymphocyte-mediated tumor killing. **(C)** Piezo2 alteration frequencies within various cancers. The mutation was the most common genetic variant of Piezo2. **(D)** The analysis of Piezo2 copy number alteration frequencies. Gene gain was the most frequent CNV of Piezo2. **(E,F)** The correlations of Piezo2 with TMB and MSI.

We accessed the biomarker relevance of Piezo2 by comparing it with well-recognized biomarkers according to their predictive power of response outcomes of immune checkpoint blockade (ICB) therapy. The results showed that Piezo2 alone had an area under the receiver operating characteristic curve (AUC) of >0.5 in 6 of the 23 ICB cohorts (Figure 9A). Although Piezo2 was not as predictive as most “star molecules” such as CD8, CD247, MSI, etc., the predictive value of Piezo2 had the same effect as B. Clonality.

We also evaluated the relationship between Piezo2 expression levels and immunotherapy in multiple cancers (Figure 9B). Our results revealed that high Piezo2 expression correlated with worse outcomes of anti-CTLA4 treatment in melanoma (ICB_Vanllen2015_CTLA-4), anti-PD-1 treatment in melanoma (ICB_Gide2019 PD-1), anti-PD-L1 treatment in bladder cancer (ICB_Mariathasan2018_PD-L1) but achieved good outcomes in kidney cancer (ICB_Miao2018_ICB). Meanwhile, analysis of the T dysfunction value in the core dataset suggested that Piezo2 played an important role in breast cancer (METABRIC) and endometrial cancer (TCGA). At the same time, we found that Piezo2-knockout was an important factor of tumor-killing mediated by NK cells in melanoma (Freeman 2019 NK) and (Kearney 2018 NK_20).

**FIGURE 10:**
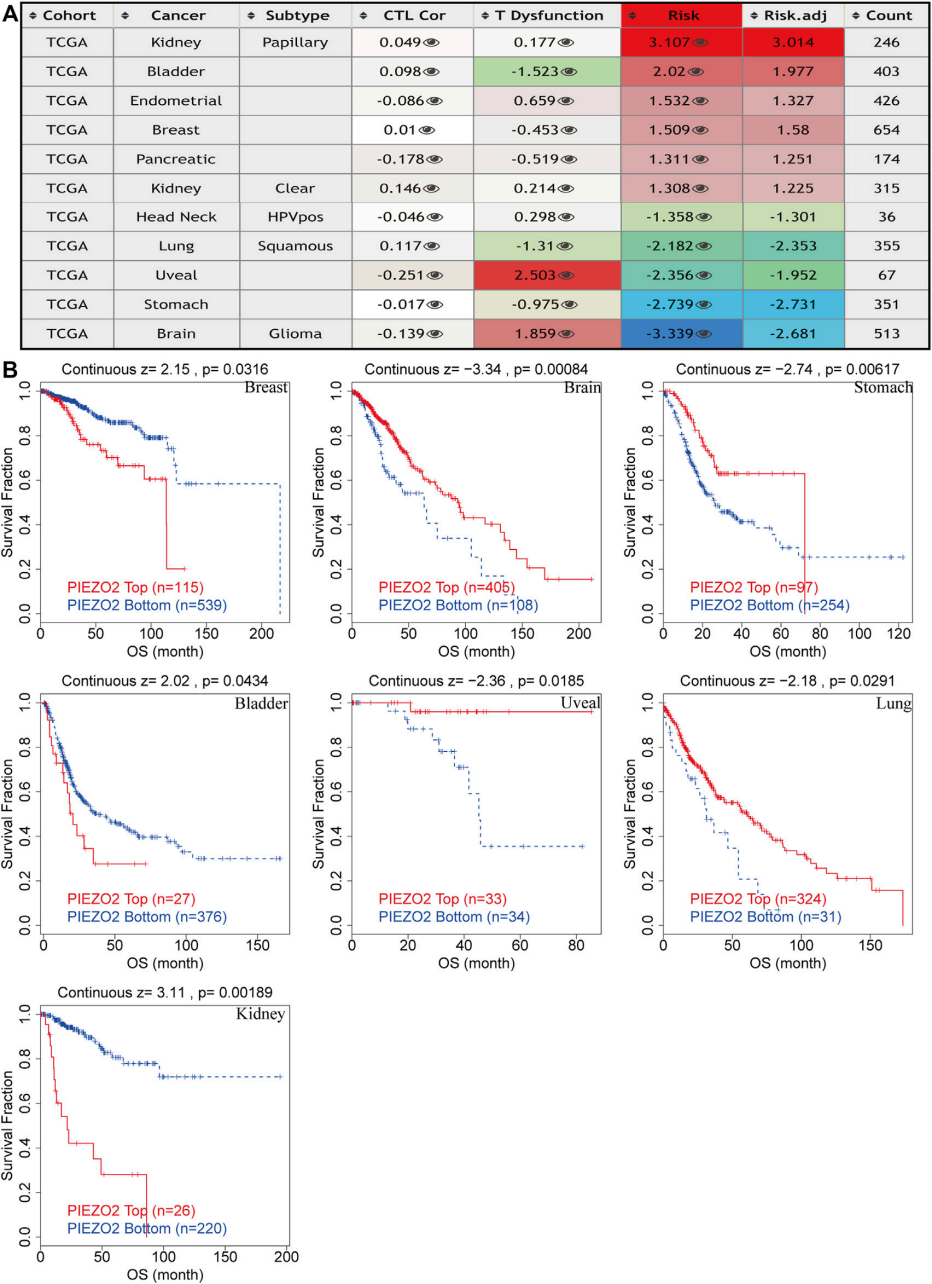
Epigenetic modification of Piezo2 influenced T cell phenotypes dysfunction, cytotoxic T-cell levels, and prognoses of different cancers. **(A)** The effects of Piezo2 methylation on T cell dysfunction, cytotoxic T-cell levels in cancers. **(B)** Kaplan-Meier curves of OS in patients with different methylation levels of Piezo2.

Subsequently, we queried the epigenetic alterations of Piezo2, including genetic alterations frequencies, copy-number alterations, MSI, and TMB. We found that gene mutation was the most common genetic variant of Piezo2, followed by gene amplification and deep deletion, while a structural variant of Piezo2 was less frequent (Figure 9C). Moreover, the results of copy-number alterations (CNV) revealed that gene gain was the most frequent CNV of Piezo2, followed by gene shallow deletion, while deep deletion and amplification were less frequent (Figure 9D). In addition, we found that Piezo2 was related to MSI in HNSC, PAAD, STAD, and UCEC. And the results revealed that Piezo2 could impact TMB in BLCA, CESC, HNSC, PAAD, etc. (Figures 9E–11F). Furthermore, we assessed the effects of Piezo2 methylation on T-cell dysfunction in multiple cancers (Figure 10A). The results suggested that hypomethylation of Piezo2 was correlated with T-cell phenotypic dysfunction. After the discovery of correlations between Piezo2 hypomethylation and T-cell dysfunction, we evaluated whether hypomethylation of Piezo2 could affect the prognosis of cancer patients. Surprisingly, hypomethylation of Piezo2 reduced survival time among patients with lung cancer, uveal cancer, stomach cancer, and brain cancer. In contrast, hypomethylation of Piezo2 was associated with a good prognosis for patients with kidney cancer, bladder cancer, and breast cancer. (Figure 10B).

### GSEA of Piezo2

Due to the ion channel properties of Piezo2, we considered whether Piezo2 could affect downstream signaling and thereby involve in the progression and metastasis of the tumor. As presented in Supplementary Figure S3, GO and KEGG analysis showed that Piezo2 was mainly enriched in cancer calcium signaling pathways. This was also in line with the calcium-gated properties of Piezo2. Surprisingly, we found that Piezo2 was also involved in the activation of immune response in OV, ACC, ESCA, LUAD, etc (Supplementary Figure S3A). Moreover, Piezo2 was enriched in antigen processing and presentation pathways (Supplementary Figure S3B). These indicated that Piezo2 may play some roles in immune processes.

## Discussion

In the occurrence of malignant tumors, especially transformation and invasion, tumor cells were subject to mechanical stresses from the surrounding environment when they crossed the basement membrane and migrated to distant sites (Mierke, 2019). Piezo2, a member of the mechanosensitive ion channel piezo family, senses cell-matrix environment and mechanical changes, then regulates cation influx to influence cell biological behavior (Coste et al., 2010). Since it was discovered in 2010 (Coste et al., 2010), the piezo family which won the Nobel Prize in Physiology or Medicine in 2021 has gradually been studied and explored. The associations between Piezo1 and cancers have been extensively studied, such as colon cancer (Sun et al., 2020), breast cancer (Luo et al., 2022), and bladder cancer (Etem et al., 2018) et al., whereases the role of Piezo2 in pan-cancer has not been well studied. Therefore, in this article, we mainly analyzed the implications of Piezo2 in the prognosis and immunotherapy of multiple cancers using the UCSC Xena database and some online analysis tools.

In this study, we interrogated that the expression levels and epigenetic alterations of Piezo2 were associated with tumor staging, the prognosis of patients, and immunotherapy for various cancers. Piezo2 was mainly expressed in the cytoplasm, located on the cell membrane and co-localized with microtubules and endoplasmic reticulum in cells. To gain more knowledge about Piezo2 function, we explored Piezo2 mRNA and protein expression differences in multiple cell lines and tissues. Piezo2 presented a similar expression level in normal tissues. In subsequent detection of tumor cell lines, we found that Piezo2 was expressed in the kidney and urinary bladder, lung, mesenchymal, and lymphoid tumor cell et al. Through the analysis of TCGA data, the results showed that Piezo2 was up-regulated in tumor tissues from CHOL, HNSC, KIRC, LIHC, PCPG, STAD, and THCA compared with normal tissues. In contrast, Piezo2 was down-regulated in tumor tissues from BLCA, COAD, GBM, KIRP, LUAD, LUSC, PRAD and READ. Huang et al. (Huang et al., 2019) also found that Piezo2 mRNA expression levels were decreased in tumor tissues of LUAD and LUSC. Subsequent functional experiments showed that loss of Piezo2 could enhance the biological behavior of tumor cells including migration and proliferation ability, which suggested that Piezo2 may play an inhibitory function in tumor progression of non-small cell lung cancer (NSCLC). Although there was no statistical difference in the down-regulation of Piezo2 in tumor tissues of BRCA in our study, its gene activity was significantly reduced in tumor tissues. Owing to the correlation between low Piezo2 expression and poor prognosis in breast cancer (Lou et al., 2019), believed that Piezo2 could be used as a prognostic indicator in breast cancer. In contrast to our study, some scholars found that Piezo2 was highly expressed in GBM (Yang et al., 2016) and BLCA (Etem et al., 2018). Additional experiments were needed to prove the expression levels of Piezo2 in different cancers. These findings indicated that Piezo2 might play different functions in different types of tumors.

Although there were correlations between Piezo2 expression and clinical stage in multiple cancers such as BRCA, KIRC, KIRP, and THCA, we found no clear trend between different stages. For patients suffering from KIRP, PAAD, and CRC, the Piezo2 expression levels were lower in patients without lymph node metastases than those with lymph node metastases. In contrast, Piezo2 expression levels were relatively low for patients with distant metastases of HNSC and KIRC. Due to the inconsistency between the correlation of Piezo2 expression and various clinical stages in different cancers, we next examined the relationship between Piezo2 expression levels and the prognosis of patients with different cancers. Cox regression analysis and Kaplan-Meier analysis indicated that high Piezo2 expression suggested a good prognosis of OS for patients suffering from KIRC, LUAD, and USC and a poor prognosis for those with KIRP and SARC. With regard to the exploration of DFS, DSS, and PFS, we found that Piezo2 seemed to be a bad indicator for patients with KIRP and PAAD, whereas a good indicator for patients with KIRC. These findings were also consistent with the previous results of Piezo2 expression levels in tumor tissues.

In a variety of tumors such as lung cancer and bladder cancer (Cao et al., 2021; Wu et al., 2021), TME was used to assess the prognosis and efficacy of immune immunotherapy. We investigated immune scores and stromal scores to determine whether there was a correlation between Piezo2 and TME. In multiple cancers, the expression levels of Piezo2 were positively correlated with the immune scores and were also positively associated with the stromal scores. The immune cells in TME played either tumor-promoting or tumor-suppressing functions in angiogenesis, cell invasion, metastasis, etc. While cytotoxic T cells played a major role in the tumor microenvironment, resting CD4^+^ T memory cells also were potential response markers for precision detection (Zhang et al., 2020). In the construction and validation of lung cancer-related prognostic genes (Zhang et al., 2021), found that resting mast cells had the potential ability to predict individualized regimens of immunotherapy. And dendritic cells were also a promising target of immunotherapy, which were recruited to the extracellular matrix, diminished T cell functions, and promoted tumor progression (Wylie et al., 2019). Our results showed associations between Piezo2 and CD4^+^ T memory cells, mast cells, and dendritic cells, suggesting that Piezo2 may involve in tumor progression by influencing immune infiltration or regulating immune cell function. Interestingly, in our findings, Piezo2 showed a positive correlation with M2 macrophages and a negative correlation with regulatory T (Treg) cells in UVM. M2 macrophages and Tregs are both immunosuppressive cells, and play inhibitory effects in tumor progression. So, there were complex interactions between Piezo2 and the anti-tumor or pro-tumor response of immune cells. Furthermore, our study explored the correlations between Piezo2 expression with immunosuppressive genes, chemokines, and chemokine receptors. These results revealed that Piezo2 was closely related to indictors of immune infiltration, and provided novel ideas for immunotherapy. Recently, DNA methylation was demonstrated to induce T-cell depletion and dysfunction and was a barrier to restoring T-cell function through ICB therapy (Ghoneim et al., 2017). Therefore, we postulated that methylation statuses of Piezo2 might drive the transcriptomic alterations of cancer cells. It was intriguing to note that the methylation status of Piezo2 could mediate T-cell dysfunction and cytotoxic T-cell levels. And hypomethylation of Piezo2 mediated a longer life span for breast cancer, bladder cancer, and kidney cancer patients and a shorter life span for brain cancer, stomach cancer, uveal cancer, and lung cancer patients. These further confirmed that Piezo2 showed a tissue-dependent manner of regulating the immune environment and affecting prognosis in patients with cancer.

Immune checkpoint inhibitor treatments, which target the PD-1/PD-L1 axis, have drawn increasing attention in tumor treatment in recent years (Makuku et al., 2021). Our results revealed that kidney cancer with high expression of Piezo2 exhibited better clinical benefits of therapy against the PD-1 and PD-L1 axis. By contrast, higher Piezo2 expression was associated with worse outcomes of anti-CTAL4 or anti-PD-L1 treatment in melanoma and bladder cancer. These suggested that Piezo2 may exhibit opposite results targeting different immunotherapy checkpoints in different tumor environments. In addition, based on the reactions of Piezo2 to ICB, we evaluated the predictive power of Piezo2 compared with several well-recognized cancer immune evasion biomarkers. Surprisingly, while the predictive power of Piezo2 was not as good as most of the classic biomarkers, it was similar to B. Clonality. Recent studies have identified an essential role for B lymphocytes against anti-tumor immunotherapy across various cancers, including CRC (Berntsson et al., 2016), breast cancer (Garaud et al., 2019), and hepatocellular carcinoma (Shi et al., 2013). Considering the associations between T cell functions and calcium influx (Freitas et al., 2018), we speculated that Piezo2 might play certain roles in orchestrating TME through regulating calcium signaling. Ca^2+^ channels had important implications in the occurrence and progression of cancers, and considerable results have confirmed the potential roles of Ca^2+^ influx in tumor growth (Cantonero et al., 2019). In many types of cancer, calcium influx has been proven to exert effects on cancer cell behavior including cell proliferation (Capiod, 2011), apoptosis (Orrenius et al., 2003), and cell death (Trump and Berezesky, 1995) et al. We found that Piezo2 was significantly associated with calcium ion transport and calcium signaling pathway which was consistent with its ion channel properties. Furthermore, analysis of KEEG and GO in cancers suggested that Piezo2 could influence the activation of the immune response, antigen processing, and presentation pathways. These findings also verified Piezo2’s considerable impact on immune responses.

The changes in gene expression patterns caused by genetic alterations were thought to transform normal cells into malignant cells through hyperplastic and immortalization (Garnis et al., 2004). The analyses of gene alteration types that were already known for tumor suppressors or oncogenes should provide more insights into the functional roles in tumor advancement and metastasis (Hanahan and Weinberg, 2000). We explored the genetic alteration types and frequencies of Piezo2 in different tumors. We found that gene mutation was the most common genetic alteration of Piezo2, while structural variant was less frequent. Consequently, analysis of CNV suggested that gene gain and shallow deletion variants had the highest frequency of Piezo2. Most copy number variants were polymorphisms and were common in healthy people, although some copy number variants were pathogenic (Zack et al., 2013). In most instances, Piezo2 maintained its original diploid morphology and occurred with mild loss of copy number and amplification to become monoploid or triploid. The gene dosage imbalances caused by CNV led to a sequence of genetic abnormalities, finally promoting cancer tumorigenesis and progression (Wong et al., 2007; Wee et al., 2018). As mentioned above, more precise predictive biomarkers were needed for immunotherapy. And several reports have suggested that TMB could predict the therapeutic effect in multiple cancers (Rizvi et al., 2015; Van Allen et al., 2015; Samstein et al., 2019). Moreover, correlations between high TMB and the ICB therapeutic benefit were observed in some cohorts, including non-small cell lung cancer treated with anti-PD-1/L1 therapy (Rizvi et al., 2015) and melanoma treated with CTLA4 inhibitors (Van Allen et al., 2015) et al. Our study confirmed that Piezo2 expression was negatively correlated with TMB in BRCA, BLCA, STAD, KIRP, and other 11 cancers. Moreover, our study also revealed correlations between Piezo2 expression and MSI. These results confirmed that Piezo2 could influence TMB and MSI in cancers and impact immunotherapy response in patients, which provided new insights to improve therapy outcomes.

Although we have extensively explored the prognostic and immunological roles of Piezo2 expression in pan-cancer, there were some limitations to our analysis. First, there may be bias and heterogeneity in multiple analyses of different databases. Therefore, the contradictory results needed further verification. Second, external validation studies were warranted to confirm the associations between Piezo2 expression and tumor prognosis. Third, it would be better to confirm the relationship between Piezo2 and immune cells through simple experiments *in vivo* or *in vitro*.

## Conclusion

To our knowledge, our study was the first to explore the roles of Piezo2 in pan-cancer. In brief, our analysis suggested that Piezo2 expression had cell-type-dependent and tissue-dependent in different types of cancer. Piezo2 expression levels were associated with the prognosis of patients with cancer. Piezo2 played underlying roles in the TME by regulating T-cell dysfunction and affecting immune-related factors. So, Piezo2 could be a novel target used for cancer treatment in the future.

## Data Availability

The original contributions presented in the study are included in the article/Supplementary Material, further inquiries can be directed to the corresponding author.
