# Supplementary material for: A pan-cancer analysis reveals the genetic alterations and immunotherapy of Piezo2 in human cancer
Source: Front Genet. 2022 Aug 4;13:918977. doi: 10.3389/fgene.2022.918977 (PMC9386142; doi:10.3389/fgene.2022.918977)
Supplement: Supplementary file 1 [file DataSheet2.PDF]

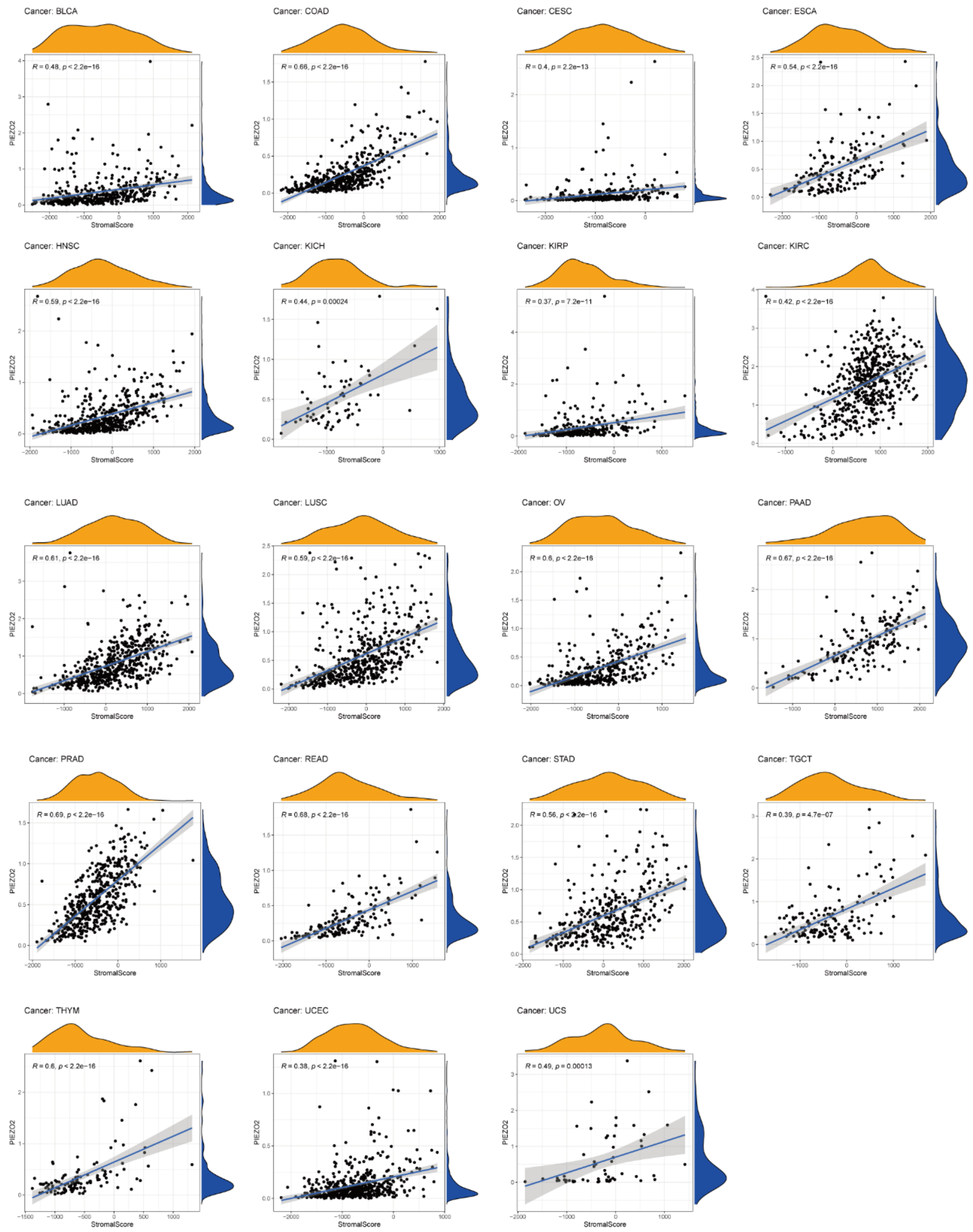

Supplementary Figure S2. The associations between Piezo2 expression and stromal scores in multiple cancers. Only results with $P < 0.05$ and $|R| > 0.3$ were considered for analysis.
