# Supplementary material for: A pan-cancer analysis reveals the genetic alterations and immunotherapy of Piezo2 in human cancer
Source: Front Genet. 2022 Aug 4;13:918977. doi: 10.3389/fgene.2022.918977 (PMC9386142; doi:10.3389/fgene.2022.918977)
Supplement: Supplementary file 2 [file DataSheet3.PDF]

A

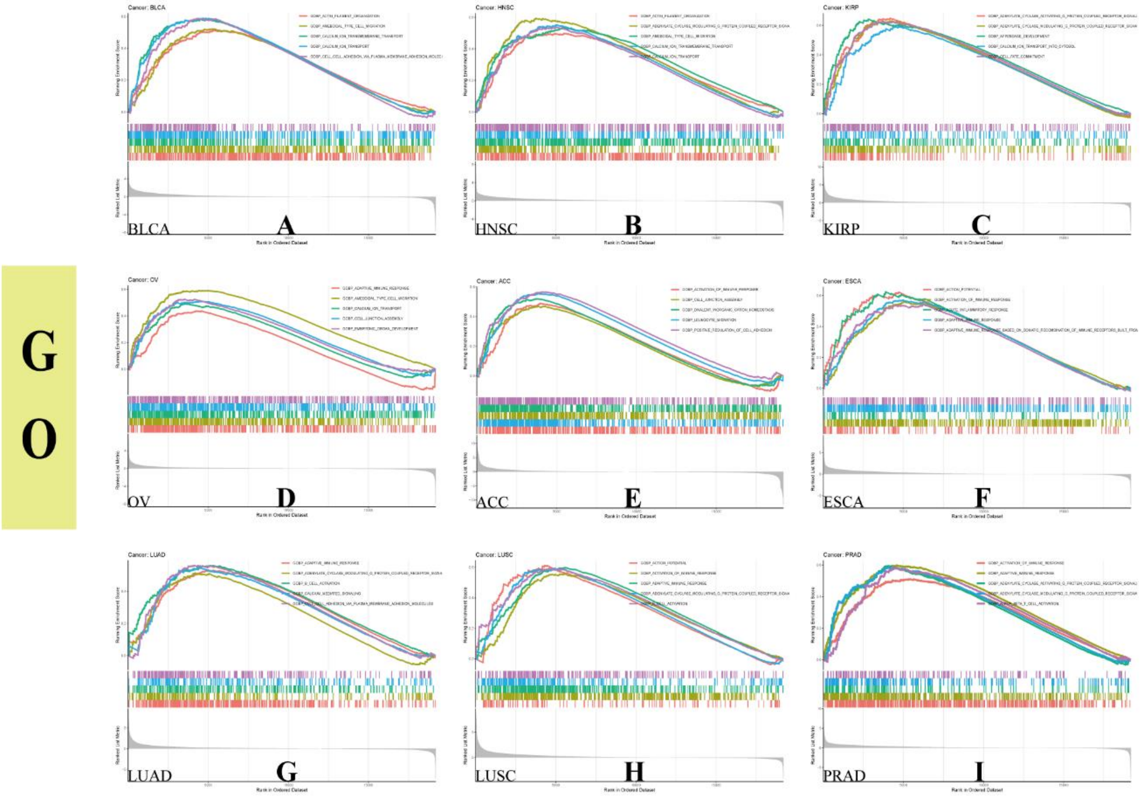
G
O
BLCA A
HNSC B
KIRP C
OV D
ACC E
ESCA F
LUAD G
LUSC H
PRAD I

B

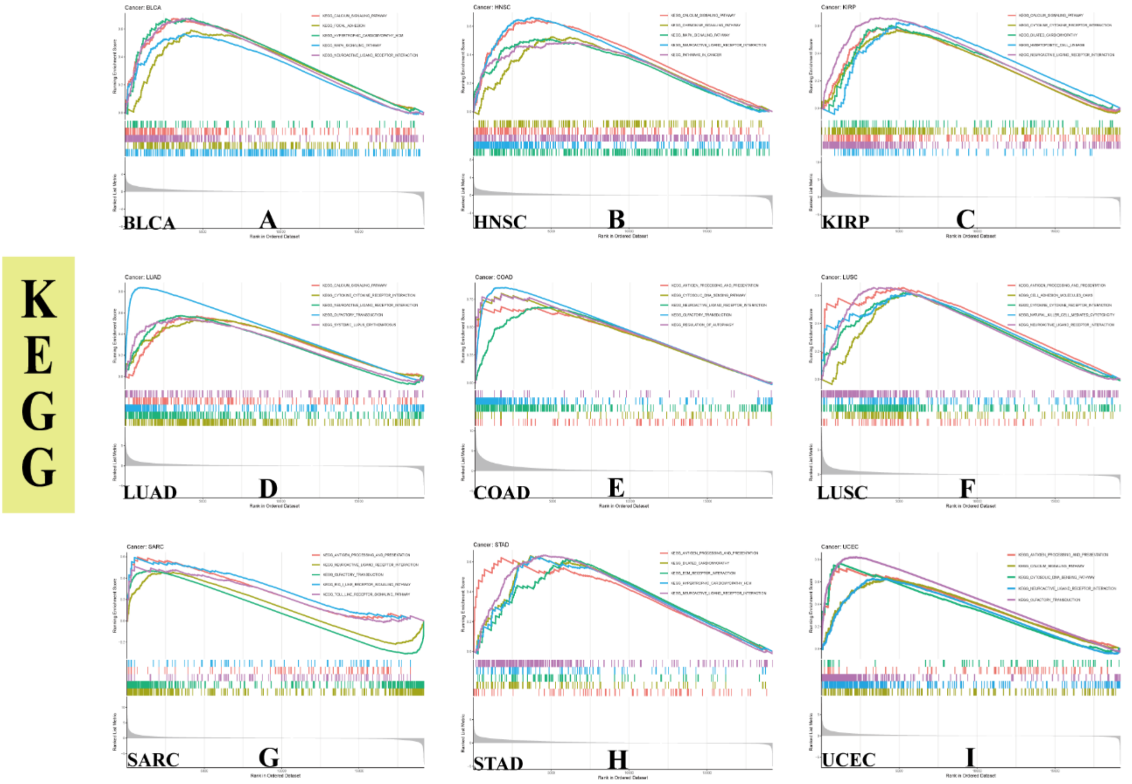
K
E
G
G
BLCA A
HNSC B
KIRP C
LUAD D
COAD E
LUSC F
SARC G
STAD H
UCEC I

Supplementary Figure S3. (A) GO analyses for Piezo2 in multiple cancers. Piezo2 was mainly enriched in calcium ion transport and activation of the immune response pathways. (B). KEGG analyses of Piezo2 in multiple cancers. Piezo2 was mainly enriched in the calcium signaling pathway, antigen processing and presentation pathway. If the peak of the curve appeared above the axis, it meant that the pathway was enriched in the high expression group of Piezo2.
