# Supplementary material for: A pan-cancer analysis reveals the genetic alterations and immunotherapy of Piezo2 in human cancer
Source: Front Genet. 2022 Aug 4;13:918977. doi: 10.3389/fgene.2022.918977 (PMC9386142; doi:10.3389/fgene.2022.918977)
Supplement: Supplementary file 3 [file DataSheet1.PDF]

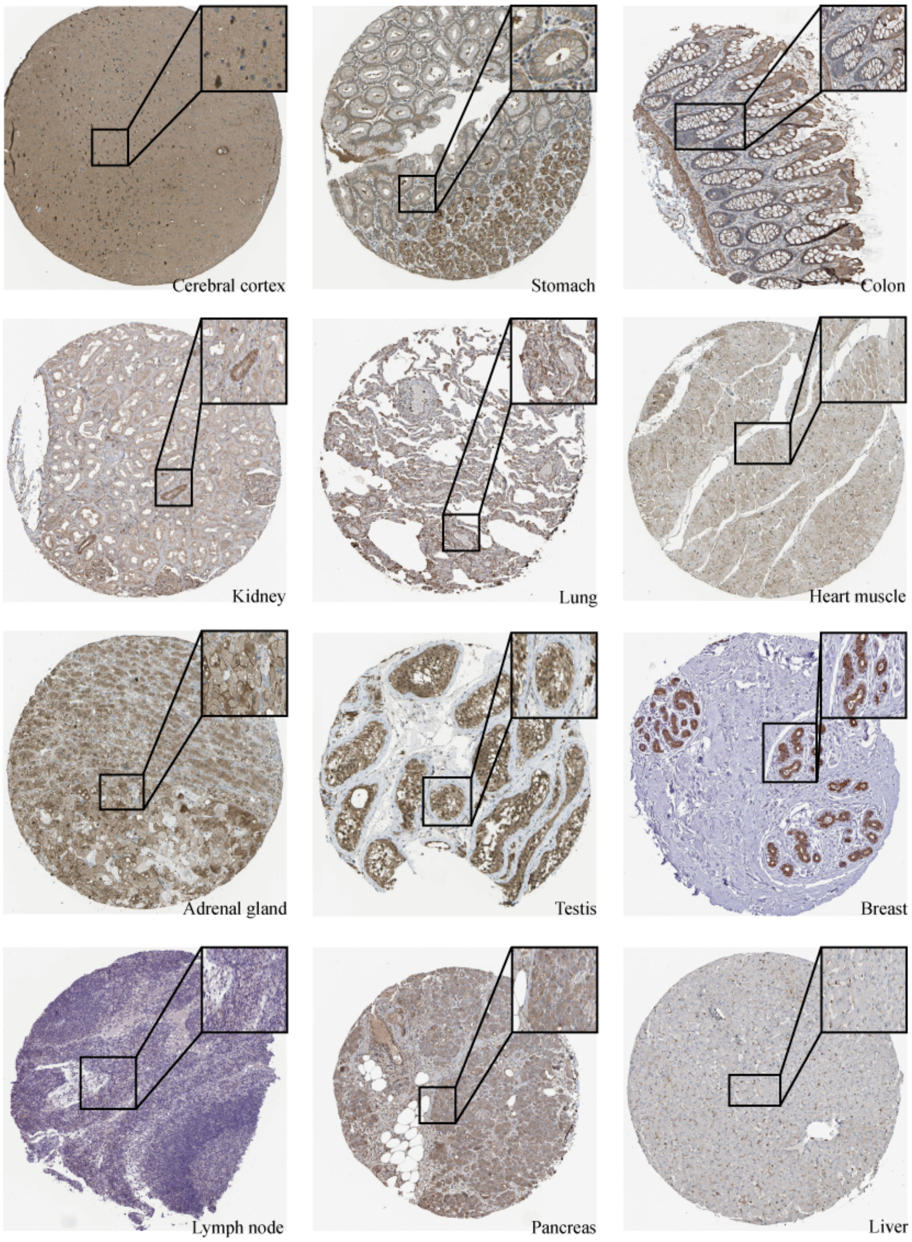


Supplementary Figure S1. The protein expression levels of Piezo2 in various human tumor tissues as adopted from the HPA database.
